# Pathophysiological Changes in Female Rats with Estrous Cycle Disorder Induced by Long-Term Heat Stress

**DOI:** 10.1155/2020/4701563

**Published:** 2020-06-17

**Authors:** GaiHong An, XueWei Chen, Chao Li, Li Zhang, MengFan Wei, JiaJun Chen, Qiang Ma, DanFeng Yang, Jing Wang

**Affiliations:** Tianjin Institute of Environmental and Operational Medicine, Tianjin 300050, China

## Abstract

High-temperature exposure is detrimental to women's reproductive health; however, the impact caused by long-term high temperature is not comprehensive, and a stable model of estrous cycle disorder induced by a high temperature is yet lacking. Herein, we aimed to establish a stable and effective model of estrous cycle disorder in female rats induced by long-term heat stress to study its physiological and pathological characteristics and explore the underlying mechanism. In the present study, female Sprague-Dawley rats with normal estrous cycles were exposed to the temperature of 38 ± 0.5°C, relative humidity (RH) of 55 ± 5% (2 h/d, 1 time/d) hot cabin at more than 90 days. Consequently, after long-term heat stress, no difference was detected in body weight and rectal temperature, but the estrus cycle was prolonged, the uterine organ index was increased, pathological changes occurred, the increase latitude of stress hormones heat shock protein 70 (Hsp70) and corticosterone (CORT) decreased, estradiol (E_2_) and luteinizing hormone (LH) levels decreased, follicle stimulating hormone (FSH) and prolactin (Prl) levels increased, gonadotropin-releasing hormone (GnRH) and thyroid hormone (T_4_) showed no difference, and insulin (INS) decreased significantly. Moreover, the mRNA expression of the sex hormone receptor in the uterus and ovary was altered. Therefore, the estrous cycle disorder in female rats can be induced by regular heat stress for 90 days, which can be considered the pioneer method. Subsequently, prominent physiological and pathological characteristics and disruption in the hypothalamic-pituitary-gonadal (HPG) axis were noted.

## 1. Introduction

High-temperature stress affects the menstrual function of career women, such as soldiers, textile workers, and steel workers, resulting in a significant increase in the incidence of abnormal menstruation. Although the effects of short-term heat exposure on female reproductive function have been studied [1], the mechanism of the impact has not been elucidated. With the increase in global temperature, the expansion of high-temperature environment and the prolonged duration of high-temperature are obvious. Also, the changes in the reproductive function of professional women in a long-term high-temperature environment need to be clarified.

Interestingly, significant gender differences are noted in the coping mechanisms of stressors [2]. Progesterone and estrogen have a direct effect on the response to stress, which varied at different stages of the estrous cycle [3]. Hitherto, only a few studies have focused on the changes in reproductive function induced by long-term heat stress. Also, the short-term heat exposure affected the estrous cycle was focused [4]. However, the disorder during the long-term exposure to high temperature was not apparent, and the physiological and pathological responses need to be elucidated. Therefore, establishing a stable and effective model of estrous cycle disorder caused by long-term heat stress is imperative to clarify the effects and mechanisms of the reproductive system in working women. Thus, the current study established the estrous cycle disorder model and studied the pathophysiological characteristics of female rats in the long-term hyperthermic environment.

The disorder of estrous cycle is often accompanied by the change of hormone levels, and the functions of hormones are accomplished by binding to hormone receptors in the ovary and uterus. Therefore, the changes in the levels of sex hormones and their receptors are important in detecting contents of estrous cycle disorder. The change of the hormone receptor mRNA level is a quantitative response to the gene function and can auxiliary support the change in sex hormone concentration. RT-qPCR has also been widely used to assess the mRNA expression level because of its advantages, such as accuracy and sensitivity [5, 6]. So, we tested the hormone receptors' mRNA expression via RT-qPCR and detected the hormone protein levels by ELISA. In addition, combined with other pathophysiologic changes and quantitated the conditions of the model, to observe the physiological and pathological responses to high temperature on the reproductive health of female animals.

## 2. Materials and Methods

### 2.1. Animals

Specific pathogen-free female Sprague-Dawley (SD) rats, weighing 200 ± 10 g, were obtained from Weitong Lihua Experimental Animal Technology Co., Ltd. (Beijing, China) and housed at 23 ± 1°C and RH 45–60% humidity under 12 h light-dark cycle with free access to food and water. The animals were fed into the cages for two days, and then vaginal cytological smears were performed at the same time every morning and evening. A total of 44 rats with regular estrous cycles were randomly divided into two groups: (1) control group, fed under the condition of standard temperature and humidity, and (2) heat stress group, each rat was exposed to heat from a small animal heat chamber (38 ± 0.5°C, RH 55 ± 5% for 2 h/day (9 : 00 to 11 : 00) for at least 90 days with food and water *ad libitum*. Blood samples from the medial canthus of 10 rats in two groups were collected at day 0, day 1, and day 90 after heat exposure. Within 24 h after the last heat exposure, blood samples of 10 rats were withdrawn from the abdominal aorta during interestrus in each group. Sera were obtained by centrifugation of the blood at 3000 rpm for 10 min and stored at -80°C; subsequently, these rats were euthanized. The uterus and ovaries of female rats were excised and stored in liquid nitrogen until further use.

All procedures relating to animal care and use were implemented in strict accordance with the National Institutes of Health Guide for the Care and Use of Laboratory animals (NIH Publications No. 8023, revised 1978) and were approved by the Ethics Review Committee of the Institute of Environmental and Operational Medicine.

### 2.2. Body Weight and Rectal Temperature Test

The body weight and rectal temperature of the animals were measured before and after 2 h heat exposure every 10 days. Digital balance (Jiangsu, Tong Jun) and thermal probe (1529 thermometer, Fluke Corporation) were used to detect the body weight and rectal temperature, respectively [7].

### 2.3. Estrous Cycle Assessment

The estrous cycle was determined according to the morphological changes in the vaginal exfoliated cells. In the experimental stage, vaginal lavage was performed daily from 8 : 00 to 9 : 00 and 20 : 00 to 21 : 00. The hematoxylin-eosin (H&E) staining was observed under a light microscope at ×10 and ×40 to detect the cellular changes. According to the proportion of the three cell types, the estrus cycle was determined. The estrous period was divided into proestrus (I), estrus (II), anaphase (III), and interestrus (IV) [8].

### 2.4. Organ Index of Uterus and Ovary Test

After the rats were sacrificed, the uterus and ovaries of the rats were excised and weighed, and the organ index was calculated according to the ultimate body weight of the rats.

(1) $$\begin{array}{c} \text{Organ indexes }\left(\%\right)=\frac{\text{Organ weight }\left(\text{g}\right)}{\text{Body weight }\left(\text{g}\right)}\times 100\% \end{array}$$

### 2.5. H&E Staining

The uterus and ovary were fixed in 4% formaldehyde for 72 h, followed by H&E staining. The histopathological changes were observed under a microscope (Nikon digital sight DS-FI2, Japan).

### 2.6. Enzyme-Linked Immunosorbent Assay (ELISA) Test

The levels of serum estradiol (E_2_), follicle-stimulating hormone (FSH), luteinizing hormone (LH), progesterone (P), prolactin (Prl), testosterone (T), Insulin (INS), thyroxine (T_4_), gonadotropin-releasing hormone (GnRH), heat shock protein 70 (Hsp70), and corticosterone (CORT) were measured using a commercial ELISA kit (USCN-LIFE™, China), according to the manufacturer's instructions.

### 2.7. qPCR Analysis

Total RNA was extracted from the uterus and ovary in rats in both groups by RNA extraction kit (TakaRa, Japan). RT-qPCR was used to detect the expression levels of estrogen receptor (ER), follicle-stimulating hormone receptor (FSHR), luteinizing hormone receptor (LHR), progesterone receptor (PR), prolactin receptor (PrlR), and testosterone receptor (TR) genes. *β*-Actin was used as the internal reference gene. The primers used for the detection of ER, FSHR, LHR, PR, PrlR, TR, and *β*-actin are described in Table 1. The expression of the target gene mRNA was analyzed by 2^-*ΔΔ*Ct^ method.

### 2.8. Statistics

All the experimental data were expressed as mean ± standard error of the mean (SEM). Statistical analyses were performed using SPSS22.0. The independent sample *t*-test as well as one-way ANOVA was used to analyze the differences between two groups and among multiple groups, respectively. *P* < 0.05 and/or *P* < 0.01 indicated statistical significance.

## 3. Results

### 3.1. Estrous Cycle Disorder Model in Female Animals Induced by High Temperature

**Figure 1:**
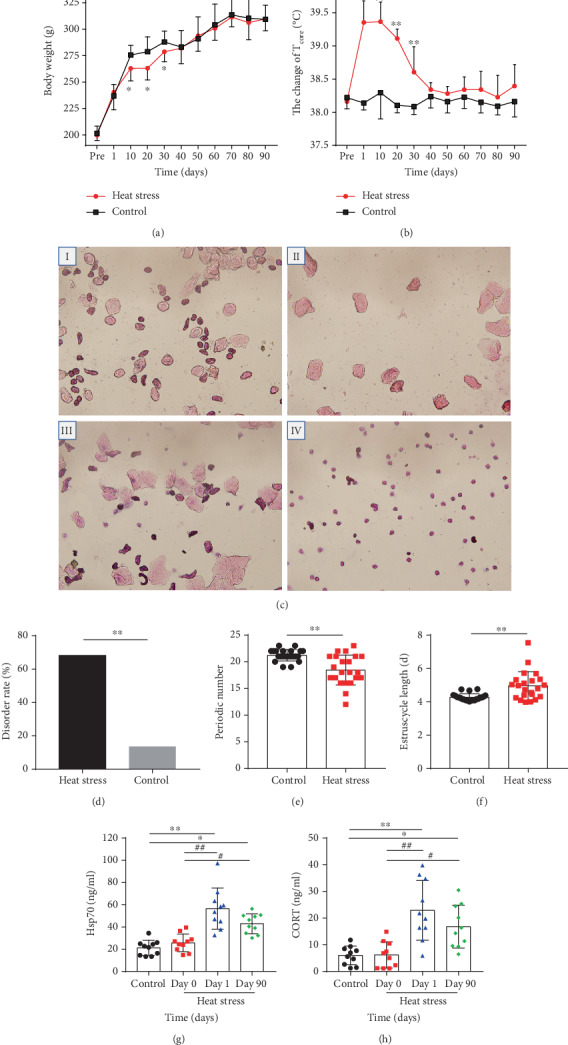
Estrous cycle disorder model in female animals induced by high temperature. (a) Change in body weight of female rats in heat stress (*n* = 10). The body weight of the rats was measured every 10 days. (b) Effects of heat exposure on *T*_core_ (*n* = 10). The *T*_core_ of the two groups of rats was determined in the rectum at 5 cm by an animal rectal thermometer every 10 days. (c) Microphotographs (10 × 40) of cellular characteristics for the identification of the estrus stage. Proestrus smear mainly consisted of nucleated epithelial cells (I); an estrus smear primarily consisted of anucleated cornified cells (II); a metestrus smear consisted of the same proportion among leukocytes anucleated cornified cells, and nucleated epithelial cells (III); and a diestrus smear primarily consisted of leukocytes (IV). (d) Effects of heat exposure on cumulative estrous cycle disorder rate (*n* = 22). (e) Effects of heat exposure on periodic numbers (*n* = 22). (f) Effects of heat exposure on estrus cycle duration (*n* = 22). (g) The content of serum Hsp70 in the heat stress group (*n* = 10). (h) The content of serum CORT in the heat stress group (*n* = 10). The protein concentration of serum Hsp70 and CORT was detected by ELISA. The blood samples from the medial canthus of two groups' rats were collected at day 0, day 1, and day 90. Values were presented as mean ± SEM. ^∗^*P* < 0.05 and ^∗∗^*P* < 0.01*vs*. the control group, ^#^*P* < 0.05 and ^##^*P* < 0.01*vs*. the heat stress group (day 0), and *^△^P* < 0.05 and *^△△^P* < 0.01*vs*. the heat stress group (day 90).

The body weight of rats in both groups continued to increase during the experiment. The body weight of the heat exposure group was unaltered from day 10 to day 20, and the weight gain was similar to that of the control group after day 30 (Figure 1(a)). The rectal temperature in the heat exposure group showed a rising trend, followed by a decline, which was significantly higher than that in the control group from day 1 to day 30 (*P* < 0.01), and no difference was observed after day 30 (Figure 1(b)). The rate of cumulative disorder in the estrous cycle in the heat exposure group was significantly higher than that of the control rats (heat exposure group: 68.18%; control group: 13.63%; *P* < 0.01) (Figures 1(c) and 1(d)). During the continuous heat exposure, the number of cycles in the heat exposure group was 18.45 ± 2.81, which was significantly lower than that in the control group (21.23 ± 1.07, *P* < 0.01) (Figure 1(e)). The normal estrous cycle lasted for 4-5 days and was extended by continuous exposure to the thermal environment (heat exposure group: 4.95 ± 0.85 days; control group: 4.27 ± 0.21days (Figure 1(f)). The Hsp70 results showed that there was no difference between the control group and day 0 of the heat stress group; it was significantly higher on day 1 and day 90 than day 0 of the heat stress group (*P* < 0.01 and *P* < 0.05, respectively) (Figure 1(g)). The level of serum CORT also was significantly higher on day 1 and day 90 than on day 0 of the heat stress group (*P* < 0.01) and the control group (*P* < 0.01). Although it was still higher on the day 90, it was significantly lower than on day 1 (*P* < 0.05) (Figure 1(h)).

### 3.2. Long-Term Heat Stress Affected the Organ Index and Histopathological Changes in Reproductive Organs in Female Rats

**Figure 2:**
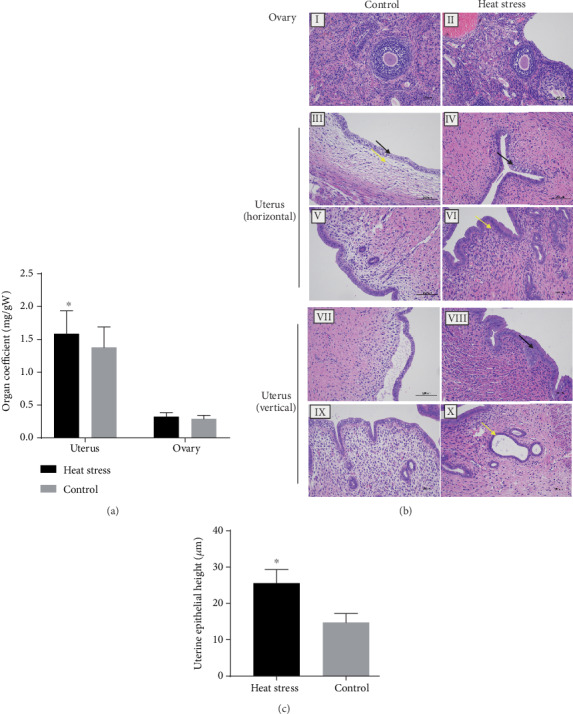
Long-term heat stress decreased the organ index of the uterus and affected the histopathology of the ovary. (a) Effect of heat exposure on the indexes of reproductive organs (*n* = 10). (b) Photomicrographs of the rat uterus and ovary by H&E staining (*n* = 3). The rat ovary (I), uterus (horizontal III and V; vertical VII and IX) in the control group. The rat ovary (II) and uterus (horizontal IV and VI vertical: VII and X) of the heat stress group. Scale bar = 100 *μ*m. (c) Graphical representation of uterine epithelial height (*n* = 10). Values are presented as the mean value ± SEM. ^∗^*P* < 0.05*vs*. the control group.

The results showed that the uterine index in the heat exposure group was significantly higher than that in the control group (*P* < 0.05), and the uterine epithelial height was similar (Figure 2(c)). However, no significant difference was detected in the ovarian index between both groups (Figure 2(a)). Also, the morphology of the ovary in the heat-exposed group did not differ from that of the control group (Figures 2(b) and 2(i), II). The uterine structure of female rats in the control group was intact (Figure 2(b), III, V, VII, and IX); also, the high columnar epithelium, fibrous cell stroma, and glands were intact and bright without any abnormality. In the heat exposure group, the uterine cavity of the female rats was narrow, and the luminal epithelial cells of the endometrium (Figure 2(b), VII, black arrow) showed an irregular morphology with epithelial cytoplasmic vacuolization (Figure 2(b), IV, black arrow) and local cell proliferation. In addition, the dilation of the uterine glands (Figure 2(b), X, yellow arrow) and a small number of neutrophils were detected in the lamina propria (Figure 2(b), VI, yellow arrow).

### 3.3. Long-Term Stress Affected Sex Hormones and Stress Hormones Levels in Female Rats

**Figure 3:**
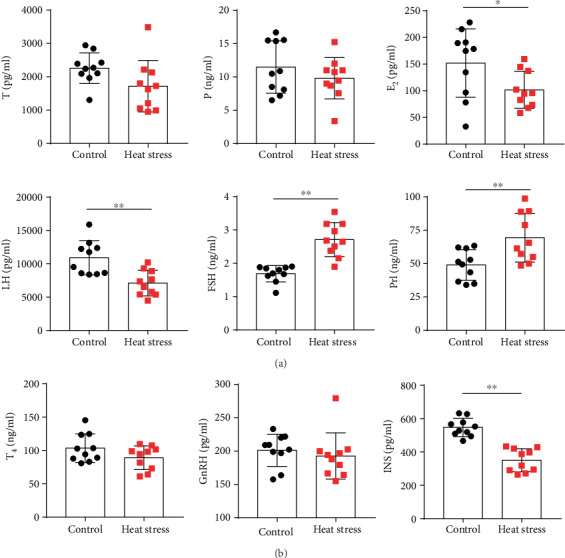
Long-term heat stress disrupted the levels of sex hormones and neuroendocrine hormones in female rats as assessed by ELISA (*n* = 10). (a) The content of serum T, P, E_2_, LH, FSH, and Prl in the two groups of rats. (b) The content of serum T_4_, GnRH, and INS in the two groups of rats. Values were presented as mean ± SEM. ^∗^*P* < 0.05 and ^∗∗^*P* < 0.01*vs*. the control group.

The results showed that the levels of serum E_2_ and LH in the heat exposure group were significantly lower than those in the heat exposure group (*P* < 0.05), while those of FSH and Prl increased significantly (*P* < 0.05). No significant difference was detected in T and P between the two groups (*P* > 0.05) (Figure 3(a)). The level of serum INS in the heat exposure group was significantly increased as compared to that in the control group; however, no difference was detected in the levels of T4 and GnRH (Figure 3(b)).

### 3.4. Gene Expression of Sex Hormone Receptors in the Uterus and Ovary of Female Rats by Long-Term Heat Stress

**Figure 4:**
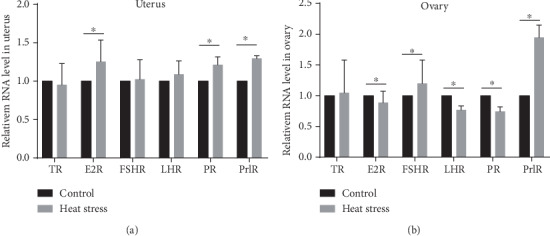
Expression of TR, ER-*α*, FSHR, LHR, PR, and PrlR genes in the reproductive organs of the two groups rats (*n* = 10). (a) Gene expression of sex hormone receptors in the uterus. (b) Gene expression of sex hormone receptors in the ovary. The level was represented as the mean value ± SEM. ^∗^*P* < 0.05*vs*. the control group.

The expression of sex hormone receptors, including TR, ER, FSHR, LHR, PR, and PrlR in the uterus and ovary, was detected by RT-qPCR. The data showed that the level of ER-*α*, PR, and PrlR in the womb of female rats in the heat exposure group was significantly higher than that in the control group (*P* < 0.05) (Figure 4(a)). In addition, the ovarian sex hormone genes, including ER-*α*, LHR, and PR were significantly decreased, while FSHR and PrlR were significantly increased in the heat exposure group as compared to the control group (*P* < 0.05 and *P* < 0.01, respectively) (Figure 4(b)).

## 4. Discussion

The impact of temperature on the fertility function in women, especially in working women, has attracted attention due to the complexity of the reproductive system of women.

However, a unified female rat model of estrous cycle disorder caused by long-term heat exposure similar to occupational exposure is yet lacking; the characteristics of estrous cycle disorder are not found to be consistent, and the related mechanism is not yet clarified. At present, most of the studies on heat stress and heat injury models focus on economic animals such as buffalo [9], dairy cow [10], pig [11], and ewe [12]. It is not suitable for the further study of the human menstrual cycle. There are few studies on heat stress in female rats, and the modeling conditions are different, and the conclusions are inconsistent [1, 4, 13, 14]. Herein, we proposed a 90-day heat stress model to redefine the temperature, duration, and frequency of heat exposure and established a stable and effective rat estrus cycle disorder model on the premise of no heatstroke. The estrous cycle is a periodic change in the nonpregnant reproductive activity of female placental mammals. The disorder in a physiological period indicates that the reproductive function of women could be damaged. Therefore, we regard estrous cycle disorder as an essential marker of changes in reproductive function in female rats induced by long-term heat stress. Previous studies have shown that short-term heat stress does not change the estrous cycle, and the disorder of the estrous cycle induced by long-term heat stress was only reported by a Russian group in 1975 [15]. In the study, female rats (140 ± 5 g) were exposed to 40°C for 12 months. The average duration of the estrous period was significantly prolonged.

Simultaneously, no significant changes were detected in the body weight and core temperature (*T*_core_) of the two groups before and after heat stress. Only in the initial 30 days of the experiment, the weight gain was decreased significantly and the *T*_core_ was increased remarkably in the heat stress group rats. Thus, body weight was a key index to reflect the nutritional status and energy metabolism of the body. The sudden decline in weight gain during acute heat exposure within 1–30 days might be related to decreased appetite and reduced food intake and energy metabolism [16], while another possibility is that heat exposure leads to increased intestinal permeability [17, 18]. Reports [19] have shown that layer chickens had a 20% reduced feed intake during hot and humid weather. The intestinal epithelial barrier in rats was damaged in acute heat exposure for 25 min (45°C, relative humidity 55%), resulting in a significant decrease in the expression of occludin, claudin, ZO-1, and JAM-A, and a significant decrease in the number of Paneth and goblet cells from 940.8 ± 8.4 to 448.8 ± 8.4 [20]. Paneth and goblet cells are important components of intestinal epithelium, and the depletion of these cells will lead to epithelial barrier defect [21]. *T*_core_ is often used to indicate the efficiency of the body thermoregulation system. Previous studies reported [22, 23] that during the heat exposure period, the *T*_core_ rose rapidly and linearly, while *T*_core_ of animals was less than the environmental heat load (*T*_e_). The thermoregulatory center dysfunction occurred when *T*_core_ reached 42°C ± 0.5°C. Typically, rat *T*_core_ > 42 ± 0.5°C is defined as a thermal injury (heat stock) marker [22, 23]. In the current study [7, 24], the temperature was limited to 38.0 ± 0.5°C, >36.0°C, and< 41.0°C, which is “compensable heat stress” [25]. The *T*_core_ was invariable between 38.0 and 39.5°C, without any obvious abnormal behavior or death in the heat stress group rats. In this study, the levels of sex hormones were disrupted in the estrous cycle disorder of female rats. The wet weight of the uterus increased, and the organ index in the experimental group was significantly higher than that in the control group. The uterine function of the female rats in the heat stress group was damaged. Previous studies reported that the organ index has a narrow range, and the increase indicates that the organ may exhibit hyperemia, edema, or hypertrophy [26]. Furthermore, under high-temperature exposure for 12 weeks, the spleen and liver of the rats atrophied and then returned to normal. The possible reason was that a series of complex immunomodulatory responses occurred after stress stimulation. On the other hand, the long-term immune response induced immune tolerance of the organs, leading to the hypertrophy of the organs [27]. Some other studies showed that estrogen causes a rapid increase in the microvascular permeability in the rodent uterus, resulting in a significant increase in stromal edema and uterine wet weight [28]. The current results showed that serum E_2_ was downregulated, and ER*-α* mRNA was upregulated in the uterus. Herein, we speculated that the growth of damp uterine pressure and partial edema might be induced by the proliferation of uterine glands, as well as the increase in the E_2_-triggered uterine microvascular permeability.

Hsp70, as a classic stress marker, is a kind of protein responds to long-term or chronic stress and can slow down the magnification response of stress-related, and it has a certain protective effect on the body. Corticosterone in rodents indicates the state of chronic stress [29]. In our study, the serum Hsp70 and corticosterone levels of female rats increased significantly after the first day of high temperature exposure and showed a downward trend after continuous heat exposure for 90 days, which may help with the rats' adaptation over time.

In addition, FSH/LH > 3 was found in the heat exposure group, indicating ovarian hyporesponsiveness or ovarian insufficiency [30]. In women, the synergistic effect of LH and FSH on ovarian follicles and granulosa cells led to an increase of estradiol in the blood, which is essential for the production of estrogen and progesterone [31]. Thus, FSH/LH is one of the clinical indexes to evaluate the ovarian reserve function. In a normal physiological state, the body can maintain FSH/LH in the normal range through self-regulation. However, under stress, the function of the hypothalamic-pituitary-gonadal (HPG) axis is affected, especially the intervention of GnRH. This disrupts the feedback regulation of the hypothalamus-pituitary-ovary (HPO) axis, which in turn, might cause a decline in the ovarian reserve function. The current results about serum LH, FSH, P, LHR, FSHR, and PR mRNA suggested that long-term heat stress leads to the dysfunction of HPO and the damage of ovarian function. Furthermore, the content of serum GnRH in the heat exposure group did not show any significant change, which could be attributed to the different stages of stress. Thus, it is suggested that the balance of the HPO axis has not been established and may be involved in the formation of estrus cycle disorders.

In hyperthermia stress, circulating blood Prl, a stress hormone, is considered to be related to the central nervous system. Its concentration reflects the activity of neurotransmitters [32] and is highly correlated to the core body temperature [33]. After 90 days of chronic heat stress, the content of serum Prl in the experimental group increased, and the expression of PrlR mRNA in the uterus and ovary was upregulated; this phenomenon was consistent with the stress state of the rats in this group.

Insulin [34] and thyroxine [35] are vital hormones on the HPO axis and essential aspects of endocrine changes under heat stress. However, the majority of the studies suggested that the concentration of insulin was increased [36], while our study (Figure 3(b)) presented the opposite result [37]. This discrepancy might be related to the degree of thermal adaption of the body following heat exposure. High temperature led to a decrease in food intake, affecting the energy balance [38]. Moreover, the low level of LH in the blood inhibited the secretion of INS, thus reducing the glucose metabolism of the body to resist the heat effect of high temperature because LH was closely related to INS. Also, we observed that the serum thyroid T_4_ concentration decreased in the heat stress group, albeit without a significant difference. Reportedly, the thyroid secretory function is decreased during heat stress, the primary metabolism is weakened, and the production of body heat is reduced to maintain the balance of heat production and heat dissipation [39]. Under acute heat stress, T4 secretion decreased [39, 40]; however, after long-term mild heat stress, the body showed thermal adaptation, and the T4 level gradually returned to the average level.

In summary, we investigated the physiological and pathological characteristics of estrus cycle disorder caused by long-term high temperature in female rats. The conditions for the model were definite, the physiological and pathological responses were obvious, and the HPG axis was disrupted. The present study would provide the experimental basis for the study of the regulatory mechanism of the estrus cycle disorder for an in-depth understanding of this field.

## Figures and Tables

**Table 1:** Primer sequences used for real-time quantitative PCR.

Gene	Forward (5′–3′)	Reverse (5′–3′)
*β*-Actin	CCTAAGGCCAACCGTGAAAA	CAGAGGCATACAGGGACAACAC
ER-*α*	GCTTATTGACCAACCTGGCAGAC	AGGATCTCCAACCAGGCACAC
FSHR	GCTGGATTTGGAGACCTGGAGA	CATGCAACTTGGGTAGGTTGGAG
LHR	AACCTGCTATACATTGAACCTGGTG	AAGGGTTCGGATGCCTGTG
PR	TCGTACAAGCATGTCAGTGGACAG	CATGGTAAGGCACAGCGAGTAGAA
PrlR	GGTGGAATCCTGGGACAGATG	CCAGATGGAAGTGTACTGCTTGCTA
TR	ATGTGGTCAAGTGGGCCAAG	ACCATCAGTCCCATCCAGGAA

## Data Availability

The data used to support the findings of this study are available from the corresponding author upon request.

## Abbreviations

RH: Relative humidity
E_2_: Estradiol
FSH: Follicle-stimulating hormone
LH: Luteinizing hormone
P: Progesterone
Prl: Prolactin
T: Testosterone
INS: Insulin
T_4_: Thyroxine
GnRH: Gonadotropin releasing hormone
HPG: Hypothalamic-pituitary-gonadal
HPO: Hypothalamus-pituitary-ovary.
